# Complement in animal development: Unexpected roles of a highly conserved pathway

**DOI:** 10.1016/j.smim.2013.04.005

**Published:** 2013-02

**Authors:** Jonathan D. Leslie, Roberto Mayor

**Affiliations:** Department of Cell and Developmental Biology, University College London, Gower Street, London WC1E 6BT, United Kingdom

**Keywords:** Complement, Development, Co-attraction, Collective cell migration, Synapse elimination, Chemotaxis

## Abstract

The complement pathway is most famous for its role in immunity, orchestrating an exquisitely refined system for immune surveillance. At its core lies a cascade of proteolytic events that ultimately serve to recognise microbes, infected cells or debris and target them for elimination. Mounting evidence has shown that a number of the proteolytic intermediaries in this cascade have, in themselves, other functions in the body, signalling through receptors to drive events that appear to be unrelated to immune surveillance. It seems, then, that the complement system not only functions as an immunological effector, but also has cell–cell signalling properties that are utilised by a number of non-immunological processes. In this review we examine a number of these processes in the context of animal development, all of which share a requirement for precise control of cell behaviour in time and space. As we will see, the scope of the complement system's function is indeed much greater than we might have imagined only a few years ago.

## Highlights

• The complement system mediates cell–cell signalling during animal development.
• In neuronal pruning, C1q and C3 mediate post-natal synapse elimination in the brain.
• C3a and C3aR drive co-attractive forces during collective cell migration.
• CL-1K and MASP direct morphogenetic movements in cranial neural crest cells.
• C3 and C5 play important roles during some forms of organ regeneration.

## 1 Introduction

**Fig. 1:**
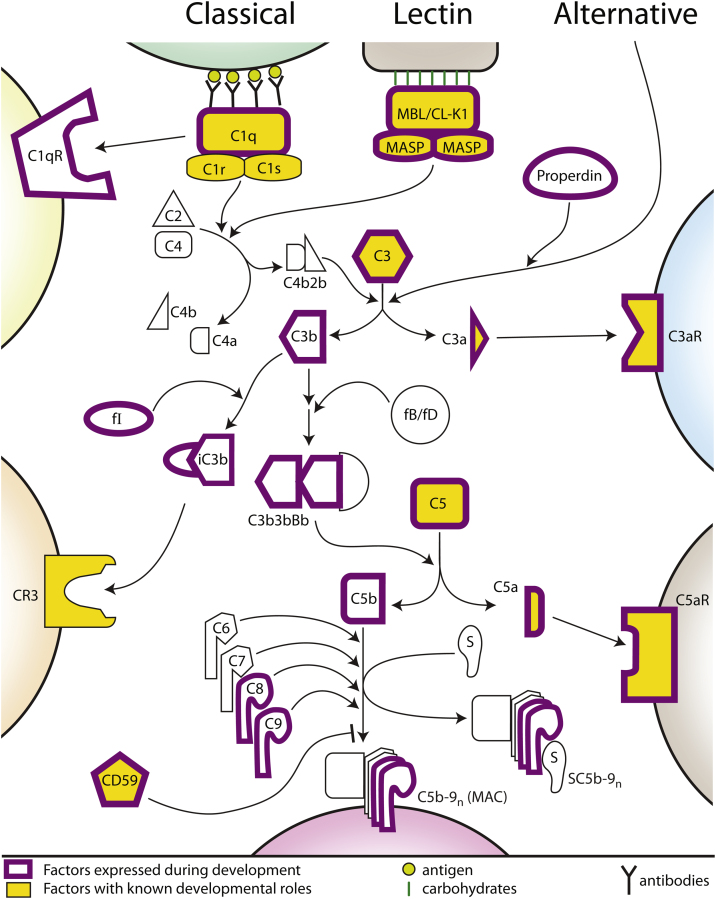
The complement pathway in development. Simplified representation of the core components of the complement system. Proteins known to be involved in developmental or regenerative processes and discussed herein are highlighted in yellow; those expressed during development or regeneration are outlined in purple. Complement system activation can occur via three separate pathways, the classical, the lectin and the alternative pathways. These converge at the level of C3 cleavage, generating C3a and C3b. C3a can signal via C3aR, while C3b production leads to the cleavage of C5. C3b can also be tagged by factorI (fI) to yield iC3b, which can signal via CR3. C5a can trigger signal transduction via C5aR, and C5b initiates the assembly of the MAC by recruiting C6, C7, C8 and a number of C9 molecules. S-protein/vitronectin (S) can also complex with C5b-9 to form a soluble form of the MAC, called SC5b-9. fB, factor B; fD, factor D.

The complement system is one of the most ancient immunological systems, with origins in some of the earliest metazoans [1–3]. At its core lies a cascade of activation steps, many of which drive proteolytic cleavages of pro-proteins into activated effectors (Fig. 1). The system is vast and a detailed description of the many components involved can be found in [4]. For the purposes of this review, we will present only the components of the system that are most relevant to the processes described below.

The complement system is activated via three independent pathways that converge on the cleavage of C3 into the fragments C3a and C3b. In the classical pathway, complement activation is triggered by C1, which is composed of C1q, C1r and C1s. Activation of C1q is often initiated by IgM or IgG clusters, and this causes the consecutive activation of C1r and C1s. C1s, in turn, cleaves C4 to generate C4a and C4b and can cleave C4b-bound C2 to produce C4b2b, which drives the activation of C3 (Fig. 1). The lectin pathway is mechanistically similar to the classical pathway: mannose-binding lectins (MBLs) recognise carbohydrates on the surfaces of pathogens, and their associated mannan-binding lectin serine peptidases (MASPs), like C1s, can cleave C4 and C2 to generate the C3 convertase C4b2b as above (Fig. 1). The alternate pathway is involved in surveillance of self versus non-self on all cells, and functionally serves to keep the immune system at an idle speed. It also drives the activation of C3 into C3a and C3b, but a detailed description of the molecules underlying alternative pathway activation is beyond the scope of this review.

Downstream of C3 cleavage C3b production triggers cleavage of C5 into C5a and C5b. C5b in turn recruits C6, C7, C8 and several C9 molecules to generate the membrane attack complex (MAC, or C5b-9_n_), which assembles in the plasma membrane of target cells, generating cytolyic pores (Fig. 1). Although osmotic lysis is one result of complement system activity, there are many other processes driven by complement system components. The anaphylatoxins C3a and C5a, which are critical for immune responses and inflammation, signal via their G-protein-coupled receptors C3aR and C5aR, respectively. Both C3a and C5a are strong chemoattractants, directing neutrophil, macrophage and monocyte migration. As we shall see, the interactions between these ligands and their receptors also drive several important developmental processes. Additionally, C3b can be tagged by factor I (fI); iC3b can bind another receptor, CR3, to trigger additional downstream effects. Even the MAC itself can have signalling roles that are different from its function as a lytic pore [5,6], as we will discuss later.

Thus, the complement system includes a vast array of ligands, receptors and regulators that can drive a large number of signalling events. While all of these are involved in immune surveillance, many are also found in other contexts. For example, some members of the complement pathway are important for neuronal elimination during brain development (reviewed in [7]). This observation is not entirely surprising: pruning of synaptic connections is an important part of the neuronal development, and elimination of neurons in later stages of development has been well documented [8,9]. Thus, it seems that development and immunology may have evolved to share the same mechanisms for ridding the body of unwanted cells.

Yet the complement system also has other developmental responsibilities that extend well beyond the realm of cell clearance. *Xenopus* embryos express a number of complement components during the early stages of development [10,11]. These early patterns of expression are not limited to amphibians: recent evidence suggests that they may be shared by other vertebrates, such as fish [12], mice [13,14] and humans [14]. These findings have led to surprising findings about how the complement pathway helps to drive morphogenetic movements during development through somewhat unexpected mechanisms [12,15–17].

In this review, we will discuss these observations in more detail and present our current understanding of many of the ways in which the complement pathway contributes to animal development. In many cases, we are only beginning to appreciate the scope this involvement. Yet as we learn more about this complex system, we begin to see a picture of a pathway whose diverse roles in non-immunological processes is indeed remarkable.

## 2 The complement pathway in development

### 2.1 Synapse elimination

For years it was believed that the immune system played no role in the central nervous system, neither during development nor in adult life. This concept of “immune privilege” was largely based on the low level of expression of immune system proteins on the surfaces of CNS cells (for example, MHC class I proteins) or the sluggish response of CNS cells to immune challenges in vivo and in vitro (reviewed in [18]). Recently, however, this notion has been challenged. With increasingly sensitive methods of detection it has become clear that molecules such as MHC class I and its effectors, cytokines and their receptors and complement pathway components are important during CNS development [14,19–23]. Of these, MHCI and cytokines are the most well-documented, with roles most often described in the context of synaptic refinement and plasticity [20,23–29]. Yet our understanding of how MHCI and cytokines signal during axonal pathfinding and synaptic refinement is far from complete, largely due to the massive degree of complexity associated with MHCI and cytokine signalling [23].

**Fig. 2:**
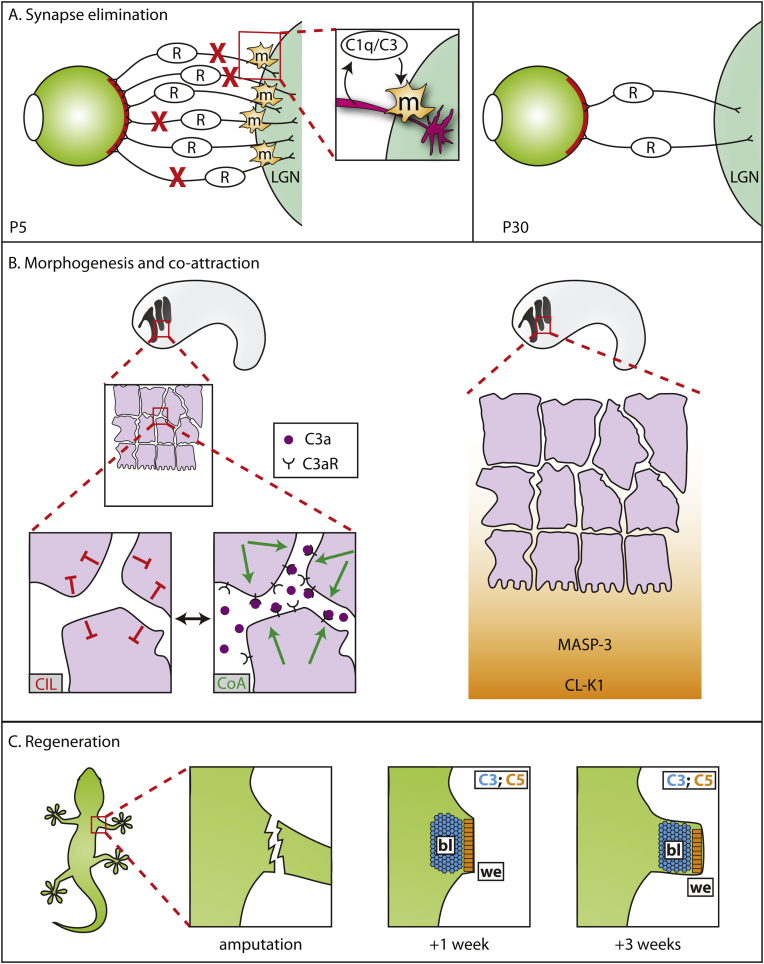
Developmental processes requiring signalling by the complement pathway. (A) Model of synaptic elimination in the mouse brain. Shortly after birth, many RGCs (R) make synaptic connections to the LGN. During the next few weeks, this number is reduced via synaptic elimination. RGCs produce C1q and C3, which is recognised by CR3-expressing microglia (m), enabling them to target a subset of the RGCs for elimination (red X), thus ensuring that the correct number of RGCs remains; (B) neural crest migration in a generic embryo. During neural crest migration (left side) repulsive forces of contact inhibition of locomotion (CIL, red) are offset by co-attractive forces (CoA, green), mediated by C3a–C3aR signalling. (Right side) Possible manner in which CL-K1–MASP interaction provides guidance cues to migrating neural crest cells; and (C) limb regeneration in the newt limb. After limb amputation, C5 protein is found on the wound epithelium (we, orange) whereas C3 is localised to the blastema (bl, blue). Surprisingly, both genes are transcribed in both structures (not shown).

More recently, the complement system has also been shown to play important roles during neural development. At birth, the mouse brain contains excessive numbers of neuronal connections between the retina and the dorsal lateral geniculate nucleus (dLGN) in the brain. This number is reduced during the first few weeks of life in a process termed synaptic elimination [30,31], and it seems that both C1q and C3 are crucial for this to occur properly (Fig. 2A). In a seminal 2007 study, Stevens and colleagues showed that complement components C1q and C3 are expressed in neonatal retinal ganglion cells (RGCs), the neurons that form these connections. This expression appears to be driven by neighbouring astrocytes by a hitherto unknown mechanism and results in the accumulation of both proteins at synapses. This accumulation is lost in older mice, indicating that its role is primarily a developmental one. In mice lacking either protein, synaptic elimination failed and excessive RGC innervation in the dLGNs was observed [13]. Thus, the complement pathway plays a role in normal brain development by mediating the elimination of unwanted neuronal connections.

If the complement system helps to eliminate unwanted neuronal connections during development, one might suppose that aberrant complement activity could be involved with neuronal degeneration during diseases or in response to injury. Indeed, it has been found that C1q, C1s and C3 are up-regulated during glaucoma, a form of eye disease characterised by RGC death [13], and that loss of C1q could ameliorate the severity of the disease [32]. Interestingly, *C1q* knock-out mice also showed signs of epilepsy resulting from enhanced excitatory synaptic connectivity [33]. Thus, the complement system appears to play a major role in the elimination of neurons, both during normal development and also in the progression of some forms of neurodegenerative disease [34]. Furthermore, complement factors also participate in other pathological neurodegenerative situations that involve elimination of neuronal connections, such as hypoxic ischaemia or spinal motoneuron injury [35].

Thus, the complement pathway is involved with synapse elimination both during normal development and in situations of disease or injury. In the case of RGCs, this appears to be mediated by microglia via CR3/C3 signalling [36]. Similar findings have been shown in studies on glaucoma [37] and brain ischaemia [35,38]. Whether the exact mechanism driving elimination in these disease states is the same as those observed during normal development remains to be determined.

### 2.2 Cell migration and morphogenesis

The findings discussed above give us important clues about non-immunological functions of complement pathway components. In both synapse elimination and immunological surveillance, the interactions between cells must be precisely controlled in time and space. One might predict, therefore, that other processes that require the careful orchestration of cell behaviour might also rely on complement-mediated signalling. An example of such a process is collective cell migration, the coordinated migration of cell clusters that has been observed during embryo development, cancer metastasis and wound healing [39–41]. We find examples of collective cell migration in a number of morphogenetic processes, such as border cell migration in *Drosophila*, lateral line development in zebrafish and neural crest migration in all vertebrates. Collective cell migration is not merely a collection of individual cells independently migrating towards the same target. Rather, it is a coordinated movement that requires cell–cell contacts to establish polarity across the cluster that, in turn, drives all of the cells within the cluster to persistently move in the desired direction [42,43]. In the neural crest, for example, all cells within a cluster will move steadily towards a chemoattractant such as the chemokine Sdf1, but isolated cells are almost completely unresponsive to Sdf1 [43]. Thus, efficient communication between neural crest cells is essential if the cells are to find their correct paths, and investigations into the mechanisms underlying this communication have revealed surprising, non-immunological roles for the complement pathway.

To understand how complement signalling affects neural crest behaviour, one must first understand a bit about the developmental roles of this extraordinary group of cells. During development, neural crest cells delaminate from the dorsal boundary of the neural tube and migrate ventrally in streams. Their route is dictated by positive and negative guidance cues that enable the cells to pioneer the formation of many structures of the adult body such as the muscle, bones and nerves in the head, neurons and glia of the peripheral nervous system and pigments in the skin, among others [44,45]. Because neural crest cells are mesenchymal, they form relatively transient cell–cell junctions with one another. Curiously, they also undergo a form of repulsion called contact inhibition of locomotion (CIL). In CIL, motile cells that collide cease their migration, repolarise and adopt a new trajectory that aims them away from the point of contact (Fig. 2B) [42,46–48]. In other words, CIL drives cells apart. How, then, can a population of cells that normally repel one another coordinate their efforts to migrate as a group?

Part of the answer comes from a counteracting force termed co-attraction (CoA) or mutual attraction between neighbour cells, and it seems that the complement pathway plays an important role in its execution. Recent work has shown that complement C3a is crucial to CoA: C3a is released by migrating neural crest cells, which also express the cognate C3a receptor (C3aR) on their surfaces (Fig. 2B) [12]. Both molecules are required for proper neural crest cell migration in vivo: removal of either molecule resulted in neural crest dispersion and loss of collective migration. Strikingly, expressing both C3a and C3aR in myeloid cells, which normally migrate as individuals, imposes collective migratory behaviour on them. Thus, C3a–C3aR-mediated signalling between neural crest cells within a cluster helps to attract cells towards one another, thereby counter-balancing the forces of CIL that serve to drive them apart (Fig. 2B). The result is a cluster of migrating cells whose collective migration is far more productive than that of any one cell as an individual [39].

How does C3a–C3aR signalling exert its effect? Normally signalling via the Rho GTPase Rac1 at the leading edge of a migratory cluster triggers the persistence of lamellipodial protrusions in the direction of migration [43,49,50]. This polarity at the front of a neural crest cluster in turn imposes directionality to the rest of the group. When C3a–C3aR signalling is blocked Rac1 activation at the leading edge of a migratory neural crest clusters is lost and the cluster fails to migrate effectively [12]. These findings echo those of Shinjyo and colleagues, who showed that C3a signalling is important in controlling the migration and differentiation of neural progenitor cells (NPCs) [51]. The authors found that C3a modulated NPC response to Sdf1 by altering levels of Erk1/2 signalling. The involvement of downstream signal transduction cascades in complement-mediated events will be discussed in more detail below (Section 3).

The development of neural crest cells and the structures that they generate is one of the more striking examples of developmental morphogenesis. Because complement-mediated signalling is critical for proper neural crest migration, one might predict that defects in complement pathway signalling might cause developmental abnormalities associated with aberrant neural crest pathfinding. Recent studies into the genetic causes of a group of human diseases have shown this to be the case.

3MC syndrome is a collection of four previously described human genetic disorders sharing similar phenotypes including defects in the formation of facial, ocular and otic structures [52,53], suggesting defects in the migration of neural crest cells. Linkage analysis revealed that 3MC syndrome is caused by mutations in two genes, *MASP1* and *COLEC11* [15,17]. *MASP1* encodes several MASP isoforms that are involved in the MBL branch of the complement pathway (Fig. 1) [54]. In innate immunity, MBL binds carbohydrates on the surfaces of bacteria. MASP proteins are associated with MBLs and trigger the activation of the complement cascade (Fig. 1) [4]. *COLEC11* (*collectin 11*) encodes the protein CL-K1, a C-type lectin that, like MBL proteins, contains a carbohydrate recognition domain [55,56] and is therefore likely to be recognised by MASP proteins. Loss of either *COLEC11* or *MASP1* in zebrafish resulted in neural crest migratory defects and resultant malformations of the cranial cartilage [15]. Additionally, CL-K1 was able to act as a chemoattractant to neural crest cells in vivo and in vitro and was able to attract HeLa cells in culture [15].

Thus, components of the MBL branch of the complement pathway aid in the guidance of neural crest cells during development (Fig. 2B). Yet exactly how this occurs on a molecular level is poorly understood. Because CL-K1 and MASP proteins are secreted and normally associated with opsonisation of pathogens, we can only speculate as to their mode-of-action in the migrating neural crest. One attractive possibility is that CL-K1-associated MASP acts as a C3 convertase, generating the release of C3a, which, in turn, facilitates co-attraction via C3aR as described above. However, many of the *MASP1* mutations in 3MC pedigrees were located in exon 12 [15,17], which encodes an alternately spliced serine protease domain; the protein product of this isoform, termed MASP3, appears to lack the ability to cleave C2, C4 or C3, suggesting that the mechanism underlying its action is independent of MBL-driven complement activation [57].

Other examples of situations in which the complement pathway aids cell migration have been reported. Kurihara and colleagues found that C5a promotes collective migration in endothelial cells during angiogenesis [58]. Complement components may also play a direct role in cell adhesion: it has been shown that myoblasts, for example, can adhere to a substrate via an interaction between integrin receptors and vitronectin (S-protein)-bound SC5b-9 complex (Fig. 1) [59]. Similarly, it appears that C3 may interact with the extracellular matrix proteins fibronectin and laminin [60,61]. However, at least in the context of neural crest migration, C3a–C3aR signalling does not exert its effect by influencing adhesion [12]. The physiological relevance of the complement system's effect on cell–matrix interaction remains to be determined.

A recent study provided an additional example of the complement pathway's role in morphogenesis. In studies examining neural tube closure, the authors found that C5a and C5aR are expressed and localised to the neural tube of developing mammals and that loss of C5aR signalling exacerbates neural tube defects associated with folic acid restriction [14]. Using both pharmacological C5aR blockade and genetic loss of C5aR, the authors observed that loss of C5aR signalling, when combined with dietary folic acid restriction, led to increased incidence of neural tube defects than folic acid restriction alone. These data indicate that C5a–C5aR signalling is involved in neurulation, but suggest that this role may be redundant with other signalling pathways and that it, therefore, may only become apparent during periods of dietary stress.

### 2.3 Regeneration

Throughout development and in adult life, cells must recognise environmental cues and respond in an appropriate way. As we have seen, the complement system plays an integral part of this process in some situations. The forces driving immune responses, morphogenesis and developmental cell fate decisions are only effective if cells are able to interpret external signals correctly. Another process that uses many of the same signals to drive morphogenesis and cell fate decisions is organ regeneration [62], and it seems that the complement pathway is heavily involved. A more detailed review of how the complement system affects regeneration appears elsewhere in this issue, but due to similarities to processes covered above we have included a short description here.

Regeneration is an extraordinary process. It involves cleaning up a wounded area, mounting a defence against possible invasion during the vulnerable state, dedifferentiation of resident cells, remodelling of the extracellular matrix (ECM), redifferentiation and, finally, growth. While the immune system is important for cleaning up the wound and defending against infection, it may also be important for other parts of the regeneration process. C3, for example, is found in myoblasts at sites of regeneration, suggesting a possible role in the differentiation of skeletal muscle [63,64].

To study possible involvement of complement system components in regeneration, Del Rio-Tsonis and colleagues turned to animals that are famous for their regenerative potential, the salamander. They found that C3 is expressed in both dedifferentiating and redifferentiating limb tissue [65]. This might indicate a possible role in development, although that notion is confounded by the authors’ observation that C3 expression is absent during normal limb development.

Might this finding reflect a role for C3 independent of its involvement in the complement cascade, or might it instead be our first hints that more of the complement cascade is involved in regenerative processes? Clues to this question have come from studies on positional identity of regenerative structures. In a screen to identify cell surface proteins whose expression varies with the proximal–distal position of the wound, da Silva and colleagues identified the newt orthologue of CD59, a negative regulator of later stages of complement activation (Fig. 1) [66]. Similarly, Kimura and colleagues showed that both C3 and C5 are involved in regeneration of urodele newt limbs (Fig. 2C) and lenses [67], although the significance of the exact spatial distribution of these proteins remains somewhat of a mystery.

Is the complement pathway's involvement in regeneration limited to urodeles or might these observations point to a more general property of the pathway? To answer that question, Mastellos and colleagues examined its regenerative involvement in a system much closer to our own: liver regeneration in mice [68]. Using a murine model for liver toxicity, they found that mice lacking C5 were severely deficient in their ability to regenerate damaged livers. In contrast to damaged livers in otherwise normal mice, damaged livers in C5-deficient mice were prone to necrosis and slow to enter the cell cycle, and both defects were rescued by the administration of exogenous C5 protein. The authors further showed that this regenerative role of C5 occurs via its proteolytic C5a fragment and the corresponding receptor, C5aR, rather than via C5b-mediated MAC assembly. These findings have since been supported by studies examining liver regeneration after partial hepatectomy, which have shown that both C3a and C5a are required for the survival of liver cells [69–72]. Interestingly, Clark and colleagues found that in the context of liver regeneration, C3 activation occurs independently of all three upstream complement pathways [72].

We have highlighted several examples of non-immunological roles of the complement pathway, and described how signalling events in the complement pathway help to drive a number of developmental processes. It is interesting to note that none of these processes appear to include the formation of the C5b-9 membrane attack complex. Yet a growing body of evidence suggests that the MAC may also have signalling roles independent of its role as a mediator of cell lysis. In the following section we will explore some of the other signalling effects of various components of the complement pathway, including the terminal MAC.

## 3 Complement-mediated signalling

As we have shown, the complement system underlies a number of developmental processes. Yet our understanding of exactly how it exerts its effects is, in many instances, poor. As we learn more about many of these processes, we begin to appreciate that complement signalling recruits a large number of signal transduction pathways (reviewed in [5]), many of which are also involved in driving developmental processes. While a comprehensive analysis of the many ways in which the complement system uses signal transduction pathways is beyond the scope of this review, it is appropriate to highlight a few of the more conspicuous examples of processes driven by the complement pathway and the signal transduction systems they employ.

Cross-talk between the complement system and other signal transduction pathways has been well-documented in the context of innate immunity and cytokine production. For example, complement and TLR signalling have been shown to converge at the level of downstream MAPK signalling via Erk1/2 and Jnk [73]. The convergence of the complement system with Erk1/2 and Jnk also appears to mediate some aspects of non-lytic complement activity. For example, C3a has been shown to mediate Sdf1-triggered Erk1/2 phosphorylation during neural progenitor cell migration and differentiation [51]. Additionally, sublytic doses of C5b-9 have been found to activate Erk and Jnk in a variety of other contexts, including protection to subsequent complement attacks [74,75] and cell cycle progression [76–78].

The ability of the complement pathway to drive cell cycle progression also appears to recruit other signalling pathway. In a murine model of kidney disease, sublytic MAC has been shown to promote proliferation and cell cycle progression via PI3K/Akt signalling [79,80]. Interestingly, this effect also appeared to have the effect of stimulating the deposition of extracellular matrix. Similarly, Shankland and colleagues have found that in cultured glomerular visceral epithelial cells, the C5b-9 complex induced DNA synthesis but had a striking negative effect on cell division [81]. Other members of the complement system also appear to have proliferative effects: C5a and C5aR can drive cell proliferation, presumably independent of MAC formation [58,82]. In some cases, the proliferative effects of the complement pathway also seem to involve the activity of RGC-32, which promotes cell cycle progression, in turn, through the activity of Akt signalling [5,83]. While the evidence linking the complement pathway to cell proliferation is compelling, whether these effects of the complement pathway have roles during normal development remains to be determined.

As a testament to the complexity of complement signalling, sublytic doses of MAC can have an opposite affect, inducing apoptosis that is driven, not by passive lysis, but rather by signalling pathways. In a murine model of kidney disease, the Wang lab has shown that sublytic MAC induces apoptosis via the activation of three pro-apoptotic molecules, IRF-1, Gadd45γ and ATF3 [84–86]. Similarly, studies by the Okada lab have shown that C5a–C5aR signalling causes apoptosis in neuroblastoma cells. This effect was associated with inward calcium currents and nuclear c-fos accumulation [87,88]. C5a has also been shown drive thymocyte and adrenal medulla cell apoptosis in sepsis models, an effect that appears to be dependent upon caspase activation [89–92]. Additionally, inhibition of C5aR in cultured mouse brain endothelial cells appeared to block nuclear factor-κB (NF-κ≡)-driven apoptosis and caspase activation [93]. Others have shown that C5b-9 can activate NF-κB in some contexts [94,95]. Whether C5aR- and C5b-9-driven NF-κB activation are related remains to be determined.

Recently, a rather unexpected role of the complement pathway has come to light. Naito and colleagues found that C1q, via C1r and C1s activation (Fig. 1), can drive canonical Wnt signalling independently of the Wnt ligand itself [96]. The authors showed that C1q binds to the Wnt receptor Frizzled, and this in turn triggers C1s-driven cleavage of the Wnt co-receptor LRP6 and the stabilisation of β-catenin, with a concomitant increase in the transcription of canonical Wnt pathway target genes. It appears that this function of complement-mediated Wnt pathway activity is largely responsible for the reduced regenerative capacity that comes with ageing: removal of C1q in muscle injury models of regeneration enabled aged mice to regenerate as if they were young. This action of C1q–C1r–C1s appears to operate independently of downstream complement pathway components, illustrating that complement signalling can feed into other signalling pathways at multiple places along the pathway.

These findings underscore the notion that we are just beginning to understand some of the varied ways that the complement system affects development. Indeed, the Wnt pathway is critical from the earliest stages of development through adulthood and old age [97,98]. It seems likely, then, that complement-driven Wnt activation, as well as other complement-mediated signalling events, occurs in a variety of developmental contexts that we have yet to uncover. We have shown several examples of convergence points between the complement pathway and other signalling pathways. While many of these have come to light via studies on disease progression, it seems likely that they may also drive other processes that occur during periods of normal health and development. Indeed, many of these signal transduction pathways are critical for driving cell behaviour and cell fate decisions throughout development and in adult life. As we learn more about the intricacies of complement action we will undoubtedly discover additional complement-driven events that are involved in development, and in so doing, will shed light on novel mechanisms driving cell–cell communication.
